# Impact of compact TiO_2_ interface modification on the crystallinity of perovskite solar cells

**DOI:** 10.1038/s41598-023-43395-1

**Published:** 2023-09-26

**Authors:** Saemi Takahashi, Satoshi Uchida, Piyankarage V. V. Jayaweera, Shoji Kaneko, Hiroshi Segawa

**Affiliations:** 1 Research Association for Technology Innovation of Organic Photovoltaics (RATO), Komaba 4-6-1, Meguro-ku, Tokyo, 153-8904 Japan; 2 Research Center for Advanced Science and Technology, The University of Tokyo, Komaba 4-6-1, Meguro-ku, Tokyo, 153-8904 Japan; 3 Department of General Systems Studies, Graduate School of Arts and Sciences, The University of Tokyo, Komaba 3-8-1, Meguro-ku, Tokyo, 153-8902 Japan; 4 SPD Laboratory, Inc., Johoku 2-35-1, Naka-ku, Hamamatsu, 432-8011 Japan

**Keywords:** Energy, Solar cells

## Abstract

The effect of TiO_2_ interfacial morphology on perovskite crystallinity was investigated by modifying the micro and nanoscale surface roughness of compact TiO_2_. While surface treatments of the compact TiO_2_ layer are recognized as effective strategies to enhance the photovoltaic performance of perovskite solar cells, the discussion regarding the crystallinity of perovskite atop TiO_2_ has been limited. In this study, we explored the impact of micro and nano scale interface morphology on perovskite crystal formation and its subsequent effects on device performance. Surprisingly, despite the absence of noticeable voids at the interface between the compact TiO_2_ and perovskite layers, the perovskite crystal morphology exhibited significant improvement following either micro or nanoscale interfacial modification. This enhancement ultimately led to improved photoconversion efficiency and reduced *I–V* hysteresis. These results emphasize the importance of underlayer surface morphology in the perovskite crystallization and suggest that the presence of grain boundaries within the perovskite layer may also contribute to *I–V* hysteresis in perovskite solar cells.

## Introduction

Organometal halide perovskite solar cell (PSC) has been attracting great attention due to its remarkable optoelectronic properties^1–5^ available with a cost-effective fabrication process. PSC has achieved a drastic improvement in a photoconversion efficiency (PCE) since it was first reported in 2009^6^, that PCE now reaches over 25%, which is comparable to that of conventional crystalline silicon photovoltaics^7–10^. Among the evolution of PSCs, a significant number of highly efficient PSC devices adopt a structure wherein a perovskite layer is deposited on an electron transport layer, and titanium oxide (TiO_2_) serves as the frequently utilized electron transport layer^11–13^. However, it has been reported that an interface between TiO_2_ and perovskite layers shows a mismatch in the junction since there is a large difference in a lattice constant between TiO_2_ and that of the perovskite crystal^14^. They also reported that *I–V* hysteresis, the phenomenon which PCE value differs depending on the scan direction, is attributed to the electrostatic capacitance component resulting from the mismatch between these interfaces^15,16^ although it is worth noting that there are other proposed origins of *I–V* hysteresis, including ionic migration of perovskite^17,18^ or the ferroelectric characteristics of perovskite^19,20^. At the same time, several studies have reported enhancements in both PCE and the mitigation of *I–V* hysteresis through interfacial modifications at the TiO_2_ and perovskite interface^21–24^. In these studies, they suggested that the surface treatment resulted in an improvement of a wettability of the TiO_2_ surface and/or an adjustment of the valence band position of TiO_2_, which thus led to an enhancement of the photo carriers generated in the perovskite layer transfer to the TiO_2_ layer.

While many scientists have examined the efficacy of surface modification at TiO_2_ and perovskite interface, there has been limited discussions concerning the influence of surface treatments on the deposited perovskite crystals in previous studies. However, the influence of underlayer surface roughness has been a subject of interest across various research fields related to thin film fabrication. In the context of organic thin film transistors, it has been observed that increased surface roughness in the gate dielectric leads to a higher density of grain boundaries, resulting in an increased presence of trap states in the semiconductor layer^25,26^. In the field of heteroepitaxy, where a crystal or thin film is grown on a substrate with a different lattice constant, it is well-documented that the quality of deposited crystals can change significantly depending on the condition of the underlayer crystal interface, potentially leading to the formation of dislocations or defects^27,28^. Although perovskite films in PSCs differ from these materials, as they are primarily fabricated using spin-coating techniques and are generally polycrystalline, it is conceivable that modifications at the interface between the TiO_2_ and perovskite layers could affect not only the junction state but also the crystallinity of the grown perovskite layer.

In this report, we examined the effect of enhancing the contact of TiO_2_ and perovskite interface on the perovskite crystallinity and subsequently evaluated the solar cell device performance. First, we focused on micro-scale roughness and attempted to improve the interfacial contact between TiO_2_ and perovskite layers by introducing a FTO substrate with a smoother surface morphology than the commonly used FTO substrate. Secondly, we attempted to eliminate gaps at the nanometer level by depositing a mesoporous layer of TiO_2_ nanoparticles that serve as scaffolds for perovskite layers on top of a dense layer of TiO_2_ and further improved the TiO_2_ and perovskite contact. In order to clarify the effect of underlayer morphology, we adopted the the simplest perovskite composition (methyl ammonium lead iodide) and the device structure without any doping or passivation to exclude any possible factors that may affect the results. Surprisingly, the morphology of the perovskite films exhibited a remarkable improvement by the surface modifications that the grain boundaries observed at the perovskite layer reduced significantly. Our study demonstrated that the surface modification of a perovskite underlayer gives a drastic impact on not only to the interfacial contact but also the perovskite morphology.

## Results and discussion

### The modification of micro scale roughness on the c-TiO_2_/perovskite interface

We first focused on a micro scale surface roughness of TiO_2_ and perovskite interface, which some studies have identified the presence of voids or gaps at the interface as a major issue that leads to a poor PCE and stability of the devices^14,21^. In this study, we adopted a FTO substrate with a smooth surface morphology as a means of improving the interfacial contact of TiO_2_ and perovskite layers. FTO substrate is a common material used as a transparent conductive oxide electrode in PSC devices though its surface typically exhibits a rough surface morphology with a scale of 0.5–1.0 μm that can exacerbate the physical gap in the interface. On the other hand, FTO substrate used in this study has a flatter surface morphology than that of the generally used FTO substrate, that the standard surface roughness parameters, arithmetical mean height (*S*_a_) and root mean square height (*S*_q_), calculated as 0.012 μm and 0.015 μm, which is both smaller than half of the values of conventional FTO (Supplementary Fig. S1, Supplementary Table S1). Thus, an introduction of the flatter FTO substrate is expected to improve the planarity of the interface, thereby reducing the likelihood of voids generates.

**Figure 1:**
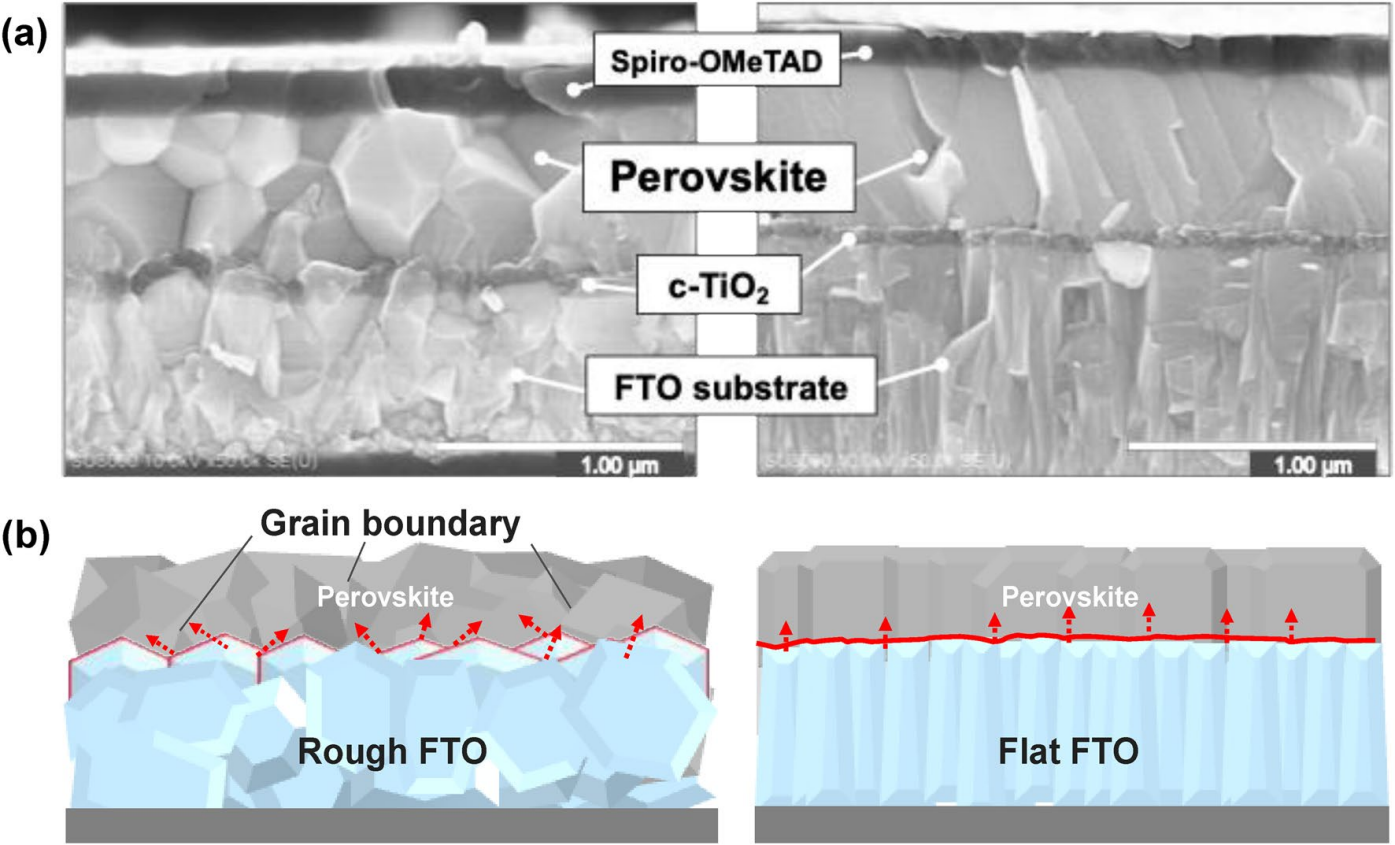
(**a**) Cross section SEM images of PSC devices with Rough FTO (left) and Flat FTO (right). A compact TiO_2_ layer is denoted as c-TiO_2_. (**b**) Schematic illustration of a possible grain growth of perovskite layer fabricated with Rough and Flat FTO substrate. Red arrows indicate growth direction of perovskite grains.

PSC device with a planer hetero junction structure was fabricated on the FTO substrate with a different surface morphology and its effect on a perovskite crystal deposited above was examined. For brevity, FTO substrate generally used in PSC devices is denoted as “Rough FTO” and FTO substrate with a flatter surface morphology is denoted as “Flat FTO” respectively. Figure 1a shows cross-sectional scanning electron microscopy (SEM) images of PSC devices prepared with the FTO substrates having a different surface roughness. Further SEM images showing a wider range of the devices are shown in Supplementary Fig. S2. According to the SEM observations, few difference was observed in a film thickness of deposited layers depending on their FTO surface textures. The compact TiO_2_, perovskite (CH_3_NH_3_PbI_3_, MAPbI_3_), Spiro-OMeTAD and Au back contact layer had an average thickness of 50 nm, 600 nm, 180 nm, and 80 nm respectively. Moreover, by focusing on the interface between the compact TiO_2_ dense layer and the perovskite layer, neither of the device had any noticeable gaps or voids at the TiO_2_ and perovskite interface, which had been proposed as a major concern by the previous studies^14,21^. This enhancement of the contact between TiO_2_ and perovskite interface may be associated with the utilization of a fast crystallization deposition, a perovskite fabrication process that drips the antisolvent during the spin coating process^29^. Application of the antisolvent dripping, followed by an immediate annealing, can expedite the crystallization of perovskite crystals. This accelerated crystallization process may result in the creation of tiny nano-sized crystals, effectively preventing the formation of a noticeable gap between the dense TiO_2_ and perovskite layers.

However, when focused on the perovskite layer itself on the other hand, there was a significant difference appeared at the perovkite microstrucuture depending on the surface morphology of the FTO substrate. The perovskite layer that was fabricated with the Rough FTO substrate exhibited grain boundaries toward different directions throughout the film with a grain size of 300–500 nm. Furthermore, since the compact TiO_2_ layer was deposited along with the rough surface morphology of the FTO substrate, it appears that the TiO_2_ layer, with a maximum thickness of 50 nm, is significantly influenced by the surface morphology of the Rough FTO. Consequently, crystal growth direction of the perovskite layer was also likely to be affected by the morphology. On the other hand, the compact TiO_2_ layer deposited on the Flat FTO had a flatter and more uniform surface in response to the morphology of the FTO substrate. Surprisingly, the perovskite layer fabricated with the Flat FTO substrate had significantly fewer grain boundaries, which predominantly oriented vertically, compared to the perovskite layer on the Rough FTO substrate. This observation clearly demonstrates the inneglisible influence of the microstructure of TiO_2_ interface on the subsequent perovksite film quality.

As previously mentioned in the introduction, researchers in the field of film fabrication have extensively explored the impact of substrate morphology on the properties of deposited films. Recent findings have highlighted notable differences in the microstructural characteristics and mechanical properties of chemically vapor-deposited Ti(C,N) coatings, despite them being produced through the same process with the only variation being the substrate's surface roughness, coarse and fine textured morphology^30^. As schematically shown in Fig. 1b, our observations will align with these reports, indicating that a rough substrate surface, particularly with the presence of macroscopic steps and edges, promotes the growth of grains that maintain the rough surface morphology. These grains undergo competitive interactions with neighboring grains during lateral growth. When grains originate on the steps of a rough substrate, their lateral expansion is restricted by adjacent grain growth, leading to the formation of grain boundaries. In contrast, when perovskite is deposited onto a flat surface, as exemplified by the Flat FTO, it tends to exhibit uniform growth along the smooth underlayer. These perovskite grains prefer vertical growth towards the substrate surface, resulting in fewer grain boundaries and a more predominant vertical orientation.

Surface morphology of the perovskite films deposited on the underlayer with a different surface morphology was also examined by the top view SEM observation shown in Supplementary Fig. S3. Both perovskite films were uniformly covered with a grain size of around 100–500 nm without any notable pinholes regardless of the surface morphology of FTO substrates. This is also comfirmed by the surface roughness measurement summarized in Supplementary Fig. S4. Although it was previously observed that the bare Rough and Flat substrates exhibited distinct differences in surface roughness as illustrated in Supplementary Fig. S1 and Supplementary Table S1, both perovskite films exhibited nearly identical surface roughness characteristics. Perovskite films fabricated on Rough FTO and Flat FTO had their surface roughness parameters calculated as 0.012 and 0.011 μm for *S*_a_ and 0.015 μm for *S*_q_, respectively and both films had a negative Kurtosis value indicating the platykutic surface. Even though the perovskite film on Rough FTO displayed coarse grains with numerous grain boundaries in the cross-sectional observation, it appears that the perovskite surface is just as smooth as that on Flat FTO, which is notably smoother than the original bare FTO substrate.

Further evaluation of each perovskite films by X-ray diffraction (XRD) measurement was operated shown in Supplementary Fig. S5. The diffraction patterns confirmed that both perovskite films have a crystal structure of an unoriented tetragonal MAPbI_3_ that is well consistent with the diffraction pattern shown by the previous report^31^. Despite the basic composition and crystal structure did not exhibit any noticeable differences, their perovskite film quality seems to vary significantly depending on their underlayer’s morphology.

**Figure 2:**
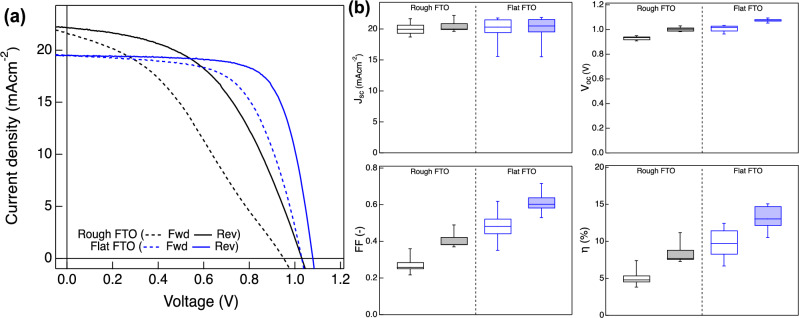
(**a**) *I–V* curves of PSC devices fabricated with Rough FTO and Flat FTO. Dashed lines and solid lines indicate forward scan (scan from 0.0 to 1.2 V) and reverse scan (scan from 1.2 to 0.0 V), respectively. (**b**) Box plots of photovoltaic parameters for 8 samples for each. A blank box correspond to the forward scan and filled box to the reverse scan.

The substantial improvement derived by the surface modification was also examined by the current–voltage (*I–V*) measurement of the solar cell devices. *I–V* curves and each photovoltaic parameters of PSC devices fabricated with Rough and Flat FTO substrates are shown in Fig. 2 and Table 1. For the evaluation of *I–V* hysteresis, the hysteresis factors^32^, defined by Eq. (1) below for the perovskite solar cells was calculated.

**Table 1:** Photovoltaic parameters of solar cells fabricated with Rough FTO and Flat FTO.

Sample	Scan direction	J_sc_ (mA cm^−2^)	V_oc_ (V)	FF (–)	PCE (%)	Hysteresis factor (–)
Rough FTO	Fwd	20.9	0.94	0.30	6.0	0.41
	Rev	21.2	1.02	0.46	10.0	
Flat FTO	Fwd	19.5	1.03	0.62	12.4	0.17
	Rev	19.5	1.08	0.72	15.1	

1 $$Hysteresis\, factor=(PC{E}_{reverse} - PC{E}_{forward}) / PC{E}_{reverse} $$

Despite both samples have the same device structure and material compositions, the introduction of Flat FTO in PSC devices also showed a clear enhancement of the photovoltaic performances. The device with Flat FTO exhibited an increase in *V*_oc_ and fill factor (FF), which resulted in an improvement of overall PCE. *J*_SC_ on the other hand, did not show a clear difference, or slightly smaller value was observed with Flat FTO which is also confirmed with the EQE measurement (Supplementary Fig. S6). This is in a good agreement with the XRD result that both perovskite films have the same composition corresponds to MAPbI_3_. Moreover, despite the mismatch between compact TiO_2_ and perovskite layer, that had been previously demonstrated as a potential cause of *I–V* hysteresis, was not observed in our devices, *I–V* hysteresis behavior also showed a difference by their FTO surface morphology.

**Figure 3:**
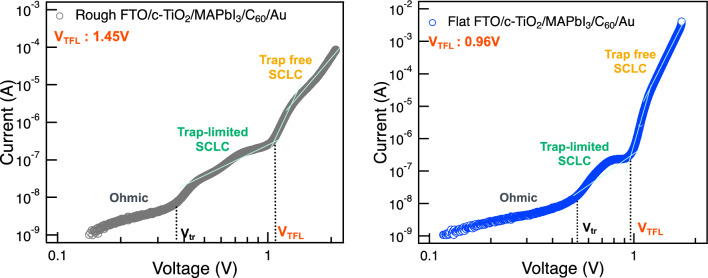
Dark *J–V* curves of electron only devices fabricated with Rough and Flat FTO.

Improvement of the device performance was further evaluated by introducing the space-charge limited current (SCLC) measurement. Electron only device was fabricated for both Rough and Flat FTO substrate with the configuration of FTO (Rough or Flat)/c-TiO_2_/perovskite/C_60_/Au and characterized for different biases. Figure 3 shows the dark *J–V* curves of the electron only devices fabricated with Rough and Flat FTO. In the low biased range, there is a linear relationship between current and voltage, characterized as the Ohmic region followed by the trap-limited SCLC region appears in the intermediate bias range. Trap density levels experience a continuous occupation, with all traps becoming filled until reaching the trap-filled limit voltage (*V*_TFL_) as bias is increased. Then, there is a surge in the current indicating the trap occupation. The trap-free SCLC region is then observable at high bias range. The onset voltage (*V*_TFL_) exhibits a linear correlation with the density of trap states^33–35^; thus, we employed *V*_TFL_ values to evaluate a trap density among the perovskite films resulting from the interface modifications. While dark *J–V* curves for full stack solar cell device did not show a distinct difference (Supplementary Fig. S7), *V*_TFL_ was characterized as 1.45 V for Rough FTO and 0.96 V for Flat FTO, indicating the reduced trap in perovskite layer by Flat FTO. This result may be induced by the perovskite layer with diminished horizontal grain boundaries previously shown in Fig. 1a, which will facilitate a carrier transportation and eventually led to the better device performance. Indeed, grain boundaries existing in the crystal have been reported to hinder a carrier transport or cause an undesired recombination of carriers, which can eventually lower the photovoltaic performance. These undesirable characteristics have been reported not only for perovskite solar cells but also for multi crystalline silicon solar cells^36,37^. deQuilettes and co-workers examined the effect of microstructure on carrier dynamics in perovskite thin films according to the photoluminescence spectroscopy^38^. They reported that grain boundaries showed lower photoluminescence and exhibited a faster nonradiative decay compared with those of bulk perovskite crystals. There is also a report that a device with potassium doped perovskite could diminish the grain boundary of perovskite layer and achieved a hysteresis free photovoltaic performance^39,40^. Their results are consistent with our suggestion that carrier recombination and/or internal resistance were successfully prevented by the elimination of grain boundaries, which may eventually lead to the improvement of solar cell performances. Our results also indicate that presence of grain boundaries in the perovskite crystal may also be a crucial factor that causes *I–V* hysteresis in PSC devices.

### The modification of nano scale roughness on the c-TiO_2_/perovskite interface

Secondly, based on the abovementioned results we further discuss the effect of TiO_2_ and perovskite interfacial contact on the deposited perovskite crystal by modifying a nano meter scale surface roughness. Application of nano structure and its influence on the subsequent film property has been reported by inorganic planer heterojunction solar cells using TiO_2_ nanoparticles^41^. Herein, we fabricated a nanoparticle TiO_2_ layer on top of the compact TiO_2_ dense layer to create a mesoscopic structured PSC device, which has been reported to have a higher efficiency than the device with the planar heterojunction structure. Using two types of FTO substrates, Rough FTO and Flat FTO, a compact TiO_2_ dense layer was deposited followed by a TiO_2_ mesoporous layer. The deposition of the perovskite layer, the hole transport layer, and the gold electrode was carried out in the same manner as the process for the planer hetero junction structure.

**Figure 4:**
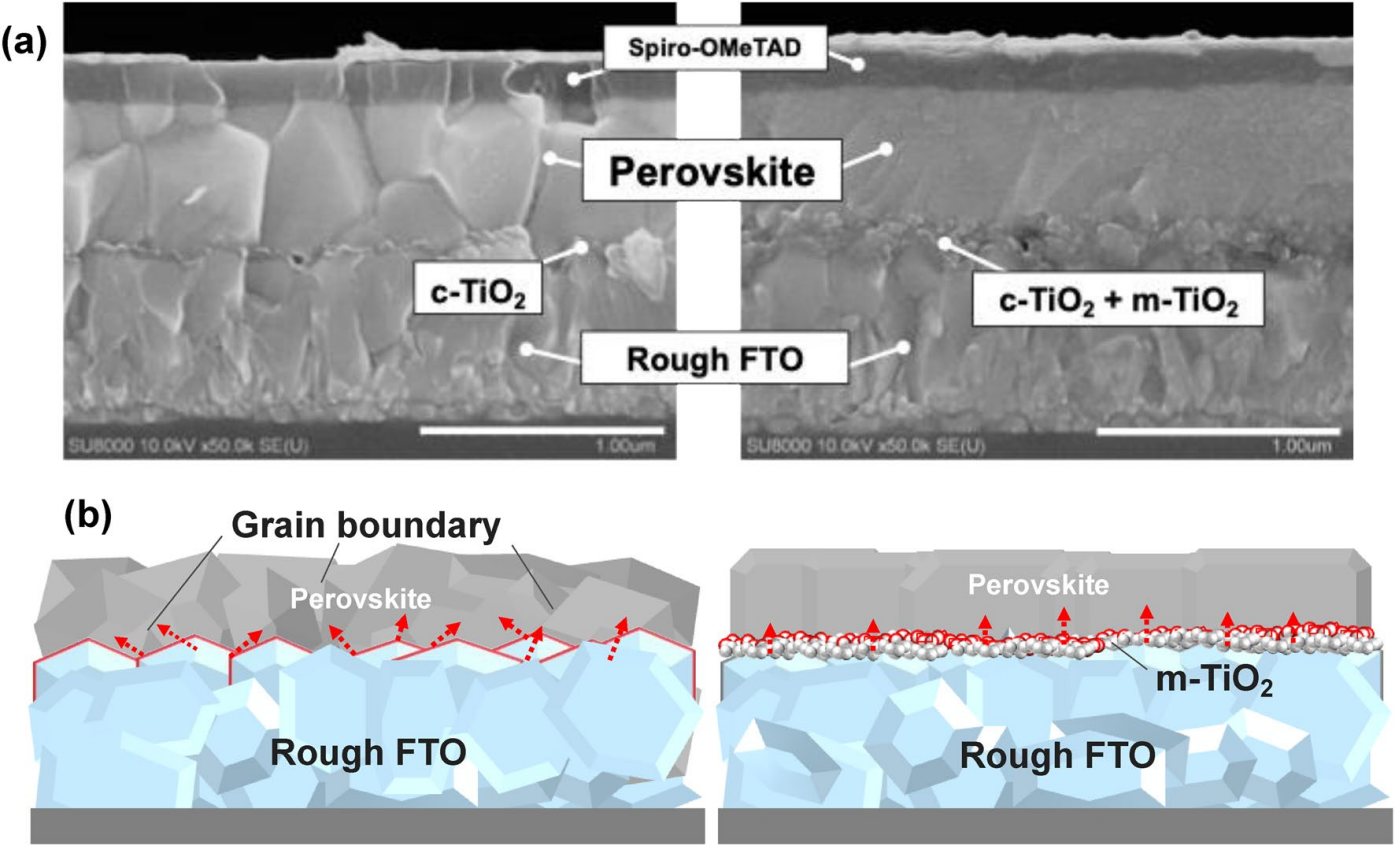
(**a**) Cross section SEM images of PSC devices with planer hetero junction (left) and mesoscopic structure (right). The caption of c-TiO_2_ indicates a compact TiO_2_ layer and m-TiO_2_ for the mesoporous TiO_2_ layer. (**b**) Schematic illustration of a possible grain growth of perovskite layer fabricated with and without mesoporous TiO_2_ layer. Red arrows indicate growth direction of perovskite grains.

Cross sectional SEM images of PSC devices fabricated with and without mesoporous TiO_2_ layer are shown in Fig. 4 (SEM images showing a wider range of the mesoscopic structure device are also shown in Supplementary Fig. S2). Both devices had almost the same film thickness for the compact TiO_2_, perovskite, Spiro-OMeTAD and Au electrode with a thickness of 50 nm, 600 nm, 180 nm, and 80 nm respectively. For the mesoscopic structure device, mesoporous TiO_2_ layer was introduced on top of the compact TiO_2_ layer with an average thickness of 100 nm. As with the result shown in Fig. 1a, while the perovskite layer fabricated with the planer hetero junction structure exhibited the notable grain boundaries toward various directions, the perovskite film fabricated on the mesoporous TiO_2_ layer appeared with the much smoother surface as well as the much less grain boundaries that their horizontal direction almost disappeared. While the device with Rough FTO presented a considerable development by the application of mesoporous TiO_2_, the device with Flat FTO did not show such a notable difference in presence of mesoporous TiO_2_ layer. However, as the Supplementary Fig. S8 shows, grain boundaries were effectively diminished, and the perovskite morphology improved slightly compared with the planer hetero junction device.

As schematicaly shown in Fig. 4b, introduction of a nano structure with mesoporous TiO_2_ layer effectively fill the gap produced from the rough surface morphology of the FTO substrates. This will enable a nano scale mechanical interlocking, which significantly reinforces the interface with the increased surface area and the total contact^42,43^. Therefore, the growth direction of the perovskite crystal was controlled, resulting in a dense film with significantly diminished grain boundaries. It has been pointed out that among the papers achieving the highly efficient PSC devices, mesoscopic structure account for a large part of the reports and thus the investigations for the reason why mesoscopic structure affords superior performance may be a key for the further improvement of PSCs^11^. While effective electron extraction facilitated by a large area contact as well as the energy level alignment between the perovskite and TiO_2_ layer have been proposed as potential benefits of mesoscopic structures, our findings also suggest an additional role of nano scale interface modification in regulating perovskite crystal growth, resulting in the formation of higher quality films with fewer grain boundaries.

According to the top view SEM observation shown in Supplementary Fig. S3, the perovskite film was uniformly covered with a grain size around 100–500 nm and there was no apparent difference in the perovskite morphology compared with other perovskite films. However, surface roughness measurement resulted in the increased value of *S*_a_ and *S*_q_ compared to that of the perovskite fabricated on Rough and Flat FTO without TiO_2_ nano particle (Supplementary Fig. S4). As the film showed a positive kurtosis value as well as the increased positive skewness value, there is a tendancy of an acute peaks at the perovskite surface, possibly due to the TiO_2_ nano particle. XRD measurement confirmed that the perovskite film fabricated on mesoporous TiO_2_ layer is a tetragonal MAPbI_3_ without any preferential orientations, which is identical to the result obtained in Rough and Flat FTO without TiO_2_ nano particle (Supplementary Fig. S5).

**Figure 5:**
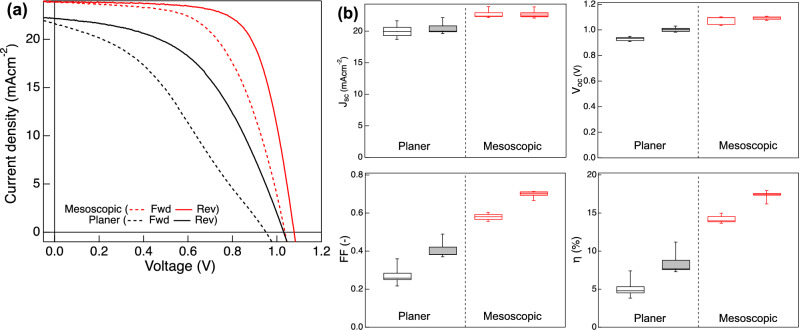
(**a**) *I–V* curves of PSC devices fabricated with planer (only c-TiO_2_) and mesoscopic (c-TiO_2_ and m-TiO_2_) structure. Dashed lines and solid lines indicate forward scan (scan from 0.0 to 1.2 V) and reverse scan (scan from 1.2 to 0.0 V), respectively. (**b**) Box plots of photovoltaic parameters for 8 samples each. A blank box correspond to the forward scan and filled box to the reverse scan.

Solar cell performance of the planer hetero junction and mesoscopic structured devices are summarized in Fig. 5 and Table 2. The device fabricated with Rough FTO, which showed *I–V* hysteresis in planer hetero junction structure previously shown in Fig. 2, achieved a significant enhancement by the introduction of the mesoporous TiO_2_ layer, that all *J*_sc_, *V*_oc_ and FF improved and PCE showed more than 1.5 times larger value than that of the planer structured devices (Fig. 5b). In addition, while the device with planer hetero junction structure showed a large hysteresis in *I–V* measurement, hysteresis factor decreased as half of the planer hetero junction structure’s value in mesoscopic structure devices. On the other hand, the device with Flat FTO which showed the improvement of PCE and *I–V* hysteresis with the planer hetero junction structure did not show a notable improvement in *I–V* hysteresis, although the photovoltaic parameters and overall PCE improved by introducing a mesoporous TiO_2_ layer (Supplementary Fig. S9). However, Rough FTO obtained a higher *J*_sc_ than that of the Flat FTO, as shown in Supplementary Table S2. This trend was also observed for the device in planer hetero junction structure as shown in Table 1. This may be due to the flat surface morphology that Flat FTO has, which reflects incident light and causes loss of absorbed light, while Rough FTO has a rough surface morphology that causes the reabsorption of reflected light and thus possible to absorb light more than the Flat FTO.

**Table 2:** Photovoltaic parameters of solar cells fabricated with planer and mesoscopic structure.

Sample	Scan direction	J_sc_ (mA cm^−2^)	V_oc_ (V)	FF (–)	PCE (%)	Hysteresis factor (–)
Planer structure	Fwd	20.9	0.94	0.30	6.0	0.41
	Rev	21.2	1.02	0.46	10.0	
Mesoscopic structure	Fwd	23.9	1.04	0.59	14.6	0.19
	Rev	23.9	1.08	0.70	18.0	

**Figure 6:**
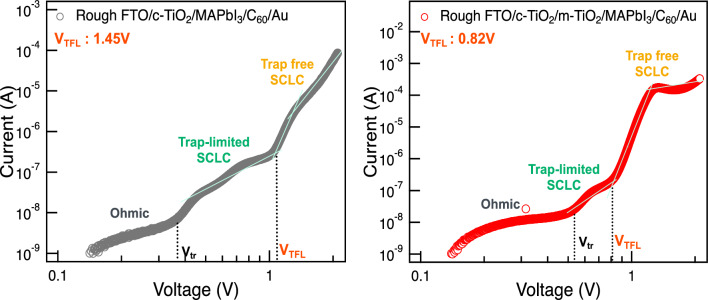
Dark *J–V* curves of electron only device fabricated with Rough FTO with and without mesoporous TiO_2_.

Enhancement of the device performance in mesoscopic structured device was also evaluated by SCLC measurement shown as Fig. 6. Electron only device was fabricated with the configuration of Rough FTO/c-TiO_2_/m-TiO_2_/perovskite/C_60_/Au. Dark *J–V* curves of the electron only devices fabricated with and without TiO_2_ nano particle were measured under the different bias, as same as the measurement shown in Fig. 3. Introduction of the TiO_2_ nano particle resulted in the decrease of trap in perovskite layer that the measured *V*_TFL_ was 0.82 V for the mesoscopic structured device while planer structured device was 1.46 V. This is lower than that of the previously shown Flat FTO results in Fig. 3, which is also in a good agreement with the better device performance of mesoscopic structured device than the planer structured device with Flat FTO. As confirmed by SEM observations, mesoscopic structure devices with both Rough and Flat FTO exhibited the improvement in perovskite morphology indicating the further efficacy of the surface treatment of TiO_2_ layer on perovskite crystallization. In addition to the enhancement in the perovskite crystallinity, an increase in the coverage area of the perovskite layer by TiO_2_ nanoparticles will lead to an efficient electron injection, which will be associated with the increased *J*_sc_ observed in the mesoscopic structured samples, and EQE shown in Supplementary Fig. S6. At the same time, since it is deposited with a thickness of around 100 nm, the TiO_2_ nanoparticles eliminated the gaps between TiO_2_ and perovskite layer within a nano meter scale, and therefore seems to obscure the impact of macro scale surface modification derived by the Flat FTO.

In summary, we examined the effect of improving the interfacial contact of TiO_2_ and perovskite on the crystallinity of the fabricated perovskite layer as well as the subsequent photovoltaic characteristics of the solar cell devices. First, we introduced a FTO substrate with a smoother surface morphology as a method to eliminate the micro scale voids between TiO_2_ and perovskite layers. Then a mesoporous TiO_2_ layer was introduced for the further nano scale modification of TiO_2_ and perovskite contact. SEM observation revealed a substantial enhancement not only in the interface between the TiO_2_ and perovskite layers but also in the overall perovskite morphology upon the incorporation of the Flat FTO substrate. Along with exploring the impact of surface treatment on the perovskite crystallinity, we also confirmed the improvement of PSC device photovoltaic characteristics as well as the *I–V* hysteresis. The observed enhancement is also confirmed by inducing the mesoporous TiO_2_ layer that the presence of grain boundaries across the entire film resulted in a noticeable decrease with a conventional Rough FTO substrate associated with the notable improvement of *I–V* hysteresis and photovoltaic characteristics. As the photovoltaic performance including *I–V* hysteresis changed in response to the perovskite morphology, the presence of grain boundaries inside perovskite layers is also likely to be responsible for the cause of *I–V* hysteresis. Our results clearly emphasize the importance of underlying surface morphology on the resulting perovskite film quality, which will contribute to the further development of the perovskite crystallization process in future.

## Methods

### Materials

All chemicals were stored in a drying room with a dew point temperature of – 30 °C and employed without having any purification. As for the transparent conductive layer, fluorine doped tin oxide (FTO) substrates were purchased from Nippon Sheet Glass Co. Ltd (NSG) and SPD laboratory Inc (SPD). They are mentioned as “Rough FTO” for the one from NSG and “Flat FTO” for the one from SPD respectively. Both FTO substrates showed an average sheet resistance of 10 Ω/□ and 80% transmittance in visible range while an average film thickness was 800 nm for Rough FTO and 1000 nm for Flat FTO. The chemicals to prepare perovskite layer, PbI_2_ (99.99%) is obtained from Kojundo Chemical Laboratory Co., LTD and MAI from Tokyo Chemical Industry (TCI). Titanium diisopropoxide bis (acetylacetonate) (75 wt% in isopropanol), lithium bis (trifluoro methanesulfonyl) imide (LiTFSI) and 4-*tert*-butylpyridine (*t*BP) are purchased from Sigma-Aldrich. TiO_2_ paste with a size of 24 nm nano particle was purchased from JGC C&C. Spiro-OMeTAD ((2,20,7,70-tetrakis-(*N,N*-di-4-methoxyphenylamino)-9,90-spirobifluorene)) is bought from Merck. All the solvents are obtained from Wako Pure Chemical Industries, LTD.

### Device fabrication

The basic device structure of fabricated PSC is FTO/compact TiO_2_/perovskite (MAPbI_3_)/Spiro-OMeTAD/Au. For the device with mesoscopic structure, mesoporous TiO_2_ was fabricated on the compact TiO_2_ layer before the perovskite layer fabrication. All samples were fabricated on same date in the same batch. As for the transparent conductive layer, FTO glasses are cleaned by a sonication in acetone and ethanol for 10 min respectively. A compact TiO_2_ layer was deposited on the cleaned FTO glass substrates by spray pyrolysis deposition with the solution of 300 µL titanium diisopropoxide bis (acetylacetonate), dispersed in 4.00 mL of ethanol. Then they were annealed at 430 °C for 15 min. For the device with the mesoscopic structure, a mesoporous TiO_2_ layer was fabricated by spin coating 24 nm TiO_2_ nano particles dispersed in ethanol with a rotation speed of 6000 rpm for 30 s. Then the sample was annealed at 550 °C for 15 min. Then a perovskite absorber layer was deposited by the fast deposition-crystallization method. A perovskite precursor solution containing 1.4 M of PbI_2_, and 1.4 M of MAI was prepared by dissolving all the powders in solvents mixing 632 µL of DMF and 71.0 µL of DMSO. After dropping the precursor solution, a two-step spin coating with a rotation speed of 1000 rpm for 10 s and 5000 rpm for 40 s was conducted to disperse the droplets. During the spin coating, 0.6 mL of chlorobenzene was quickly dropped 22 s after the beginning of the program. The as-spun films were annealed at 70 °C for 1 min followed by the further anneal at 100 °C for 30 min. Then a hole transport layer was spin coated using a solution of Spiro-OMeTAD (72.3 mg/mL) in chlorobenzene (1.00 mL). To increase conductivity, LiTFSI (520 mg/mL in acetonitrile, 17.5 µL) and *t*BP (26.8 µL) were added to the solution. The solution was spin coated with a rotation speed of 4000 rpm for 30 s and annealed for 10 min at 70 °C. At last, Au electrode with the thickness of around 80 nm was deposited by a thermal evaporation, and the area of the Au contact was more than 0.18 cm^2^. All processes were performed in the drying room and fabricated devices were stored in the dry room for a day before the measurements.

### Device evaluation

The current–voltage (*I–V*) curves were measured and recorded under AM 1.5G illumination (100 mW/cm^2^) with a 450 W xenon light source (YSS-80A; Yamashita Denso Co., Ltd., Japan). The light source was calibrated using a silicon photodiode of BS-520 (Bunkoukeiki Co., Ltd., Japan). Scan speed of the measurement was fixed as 100 mV/s. The EQE spectrum was recorded with an EQE system (CEP-2000MLQ, Bunkoukeiki Co., Ltd.) in the DC mode without any voltage bias. A black mask was used to confirm the device photoactive area of 0.18 cm^2^ during the *I–V* and EQE measurements. Surface and cross section morphology was observed using a field emission scanning electron microscope (SEM, SU 8000, Hitachi). X-ray diffraction (XRD) measurement was carried out on a Bruker D8 Discover with Cu K-alpha radiation (*λ* = 0.15406 nm). Surface roughness measurement was operated with a laser microscope (OLYMPUS, LEXT OLS4000) for a sample area of 258 μm × 258 μm.

### Supplementary Information

Supplementary Information.

## Data Availability

All data generated or analyzed during this study are included in this published article (and its Supplementary Information files).

## References

[1] Green MA, Ho-Baillie A, Snaith HJ (2014). The emergence of perovskite solar cells. Nat. Photonics.

[2] Stranks SD (2013). Electron-hole diffusion lengths exceeding 1 micrometer in an organometal trihalide perovskite absorber. Science.

[3] Dong Q (2015). Electron-hole diffusion lengths > 175 mm in solution-grown CH_3_NH_3_PbI_3_ single crystals. Science.

[4] Giorgi G, Fujisawa JI, Segawa H, Yamashita K (2013). Small photocarrier effective masses featuring ambipolar transport in methylammonium lead iodide perovskite: A density functional analysis. J. Phys. Chem. Lett..

[5] Yin WJ, Shi T, Yan Y (2015). Superior photovoltaic properties of lead halide perovskites: Insights from first-principles theory. J. Phys. Chem. C.

[6] Kojima A, Teshima K, Shirai Y, Miyasaka T (2009). Organometal halide perovskites as visible-light sensitizers for photovoltaic cells. J. Am. Chem. Soc..

[7] The National Renewable Energy Laboratory (NREL). *Best Research-Cell Efficiency Chart*. https://www.nrel.gov/pv/cell-efficiency.html. (Accessed 26 Jan 2022).

[8] Kim G (2020). Impact of strain relaxation on performance of a-formamidinium lead iodide perovskite solar cells. Science.

[9] Yoo JJ (2021). Efficient perovskite solar cells via improved carrier management. Nature.

[10] Jeong J (2021). Pseudo-halide anion engineering for α-FAPbI_3_ perovskite solar cells. Nature.

[11] Nakazaki J, Segawa H (2018). Evolution of organometal halide solar cells. J. Photochem. Photobiol. C Photochem. Rev..

[12] Jena AK, Kulkarni A, Miyasaka T (2019). Halide perovskite photovoltaics: Background, status, and future prospects. Chem. Rev..

[13] Shahiduzzaman M (2021). The benefits of ionic liquids for the fabrication of efficient and stable perovskite photovoltaics. Chem. Eng. J..

[14] Cojocaru L (2015). Temperature effects on the photovoltaic performance of planar structure perovskite solar cells. Chem. Lett..

[15] Cojocaru L (2015). Origin of the hysteresis in I–V curves for planar structure perovskite solar cells rationalized with a surface boundary-induced capacitance model. Chem. Lett..

[16] Cojocaru L (2017). Simulation of current-voltage curves for inverted planar structure perovskite solar cells using equivalent circuit model with inductance. Appl. Phys. Express.

[17] Eames C (2015). Ionic transport in hybrid lead iodide perovskite solar cells. Nat. Commun..

[18] Calado P (2016). Evidence for ion migration in hybrid perovskite solar cells with minimal hysteresis. Nat. Commun..

[19] Frost JM (2014). Atomistic origins of high-performance in hybrid halide perovskite solar cells. Nano Lett..

[20] Chen HW, Sakai N, Ikegami M, Miyasaka T (2015). Emergence of hysteresis and transient ferroelectric response in organo-lead halide perovskite solar cells. J. Phys. Chem. Lett..

[21] Cojocaru L (2015). Surface treatment of the compact TiO_2_ layer for efficient planar heterojunction perovskite solar cells. Chem. Lett..

[22] Murakami TN (2017). Adjustment of conduction band edge of compact TiO_2_ layer in perovskite solar cells through TiCl_4_ treatment. ACS Appl. Mater. Interfaces.

[23] Shahiduzzaman M (2018). Compact TiO_2_/anatase TiO_2_ single-crystalline nanoparticle electron-transport bilayer for efficient planar perovskite solar cells. ACS Sustain. Chem. Eng..

[24] Shahiduzzaman M (2019). Low-temperature-processed brookite-based TiO_2_ heterophase junction enhances performance of planar perovskite solar cells. Nano Lett..

[25] Sun X, Di CA, Liu Y (2010). Engineering of the dielectric-semiconductor interface in organic field-effect transistors. J. Mater. Chem..

[26] Geiger, M. *et al.* Effect of the degree of the gate-dielectric surface roughness on the performance of bottom-gate organic thin-film transistors. *Adv. Mater. Interfaces* **7**, (2020).

[27] Wu P (2012). Lattice mismatch induced nonlinear growth of graphene. J. Am. Chem. Soc..

[28] Kinjo R, Kawayama I, Murakami H, Tonouchi M (2013). Thickness dependence of dielectric characteristics of SrTiO_3_ thin films on MgAl_2_O_4_ substrates. Adv. Mater. Phys. Chem..

[29] Xiao M (2014). A fast deposition-crystallization procedure for highly efficient lead iodide perovskite thin-film solar cells. Angew. Chem..

[30] Wächtler C, Wüstefeld C, Šíma M, Pikner J, Rafaja D (2023). Influence of the substrate treatment on the microstructure and properties of chemical vapour deposited Ti(C, N) coatings. Surf. Coat. Technol..

[31] Stoumpos CC, Malliakas CD, Kanatzidis MG (2013). Semiconducting tin and lead iodide perovskites with organic cations: Phase transitions, high mobilities, and near-infrared photoluminescent properties. Inorg. Chem..

[32] Li Z (2017). Acid additives enhancing the conductivity of spiro-OMeTAD toward high-efficiency and hysteresis-less planar perovskite solar cells. Adv. Energy Mater..

[33] Shi D (2015). Low trap-state density and long carrier diffusion in organolead trihalide perovskite single crystals. Science.

[34] Lampert MA (1956). Simplified theory of space-charge-limited currents in an insulator with traps. Phys. Rev..

[35] Lampert MA, Rose A, Smith RW (1959). Space-charge-limited currents as a technique for the study of imperfections in pure crystals. J. Phys. Chem. Solids.

[36] Grovenor CRMM (1985). Grain boundaries in semiconductors. J. Phys. C Solid State Phys..

[37] Long R, Liu J, Prezhdo OV (2016). Unravelling the effects of grain boundary and chemical doping on electron-hole recombination in CH_3_NH_3_PbI_3_ perovskite by time-domain atomistic simulation. J. Am. Chem. Soc..

[38] DeQuilettes DW (2015). Impact of microstructure on local carrier lifetime in perovskite solar cells. Science.

[39] Tang Z (2017). Hysteresis-free perovskite solar cells made of potassium-doped organometal halide perovskite. Sci. Rep..

[40] Tang Z (2018). Modulations of various alkali metal cations on organometal halide perovskites and their influence on photovoltaic performance. Nano Energy.

[41] Chen J (2019). Preferentially oriented large antimony trisulfide single-crystalline cuboids grown on polycrystalline titania film for solar cells. Commun. Chem..

[42] Dubrovskiǐ VG, Cirlin GE (2005). Growth kinetics of thin films formed by nucleation during layer formation. Semiconductors.

[43] Liu Z (2017). High-adhesion stretchable electrodes based on nanopile interlocking. Adv. Mater..

