# Supplementary material for: Impact of compact TiO2 interface modification on the crystallinity of perovskite solar cells
Source: Sci Rep. 2023 Sep 26;13:16068. doi: 10.1038/s41598-023-43395-1 (PMC10522660; doi:10.1038/s41598-023-43395-1)
Supplement: Supplementary file 1 — Supplementary Information. [file 41598_2023_43395_MOESM1_ESM.pdf]

Supplementary information

Impact of Compact TiO_2 Interface Modification on the Crystallinity of Perovskite Solar Cells

Saemi Takahashi[1,3], Satoshi Uchida[2*] Piyankarage V. V. Jayaweera[4], Shoji Kaneko[4] and Hiroshi Segawa[1,2,3*]

[1]Research Association for Technology Innovation of Organic Photovoltaics (RATO), Komaba 4-6-1, Meguro-ku, Tokyo 153-8904, Japan

[2]Research Center for Advanced Science and Technology, The University of Tokyo, Komaba 4-6-1, Meguro-ku, Tokyo 153-8904, Japan

[3]Department of General Systems Studies, Graduate School of Arts and Sciences, The University of Tokyo, Komaba 3-8-1, Meguro-ku, Tokyo 153-8902, Japan

[4] SPD Laboratory, Inc., Johoku 2-35-1, Naka-ku, Hamamatsu 432-8011, Japan

* Corresponding author e-mail: uchida@rcast.u-tokyo.ac.jp, csegawa@mail.ecc.u-tokyo.ac.jp

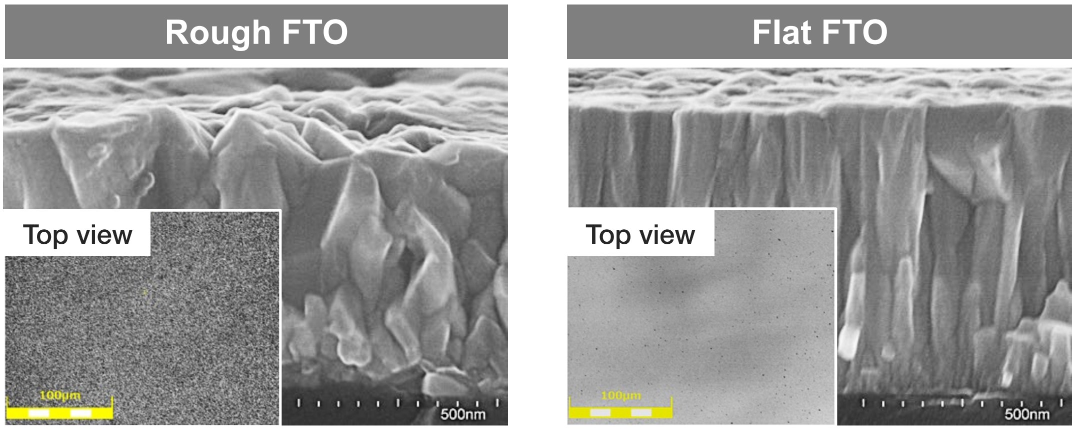

Fig. S1 Cross section and top view SEM images of Rough and Flat FTO substrates.

Table. S1 Surface roughness parameters of Rough and Flat FTO measured by a laser microscope.

	Rough FTO	Flat FTO
Arithmetical mean height (S_a)	0.028 μm	0.012 μm
Root mean square height (S_q)	0.039 μm	0.015 μm
Kurtosis (S_{sk})	-0.71	-0.33
Skewness (S_{ku})	13.75	3.78
Maximum peak height (S_p)	0.91 μm	0.24 μm
Maximum pit depth (S_v)	1.68 μm	0.15 μm
Maximum height (S_z)	2.59 μm	0.39 μm

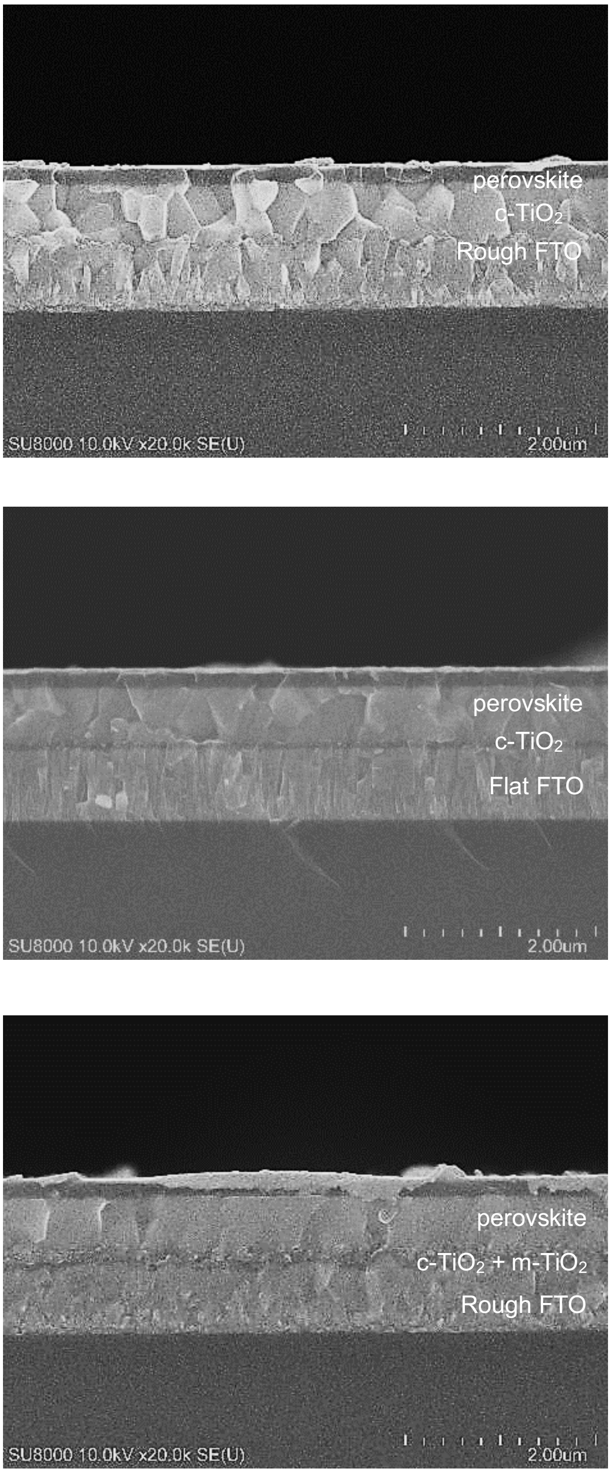

Fig. S2 Cross section SEM images of solar cell devices observed with lower magnitude.

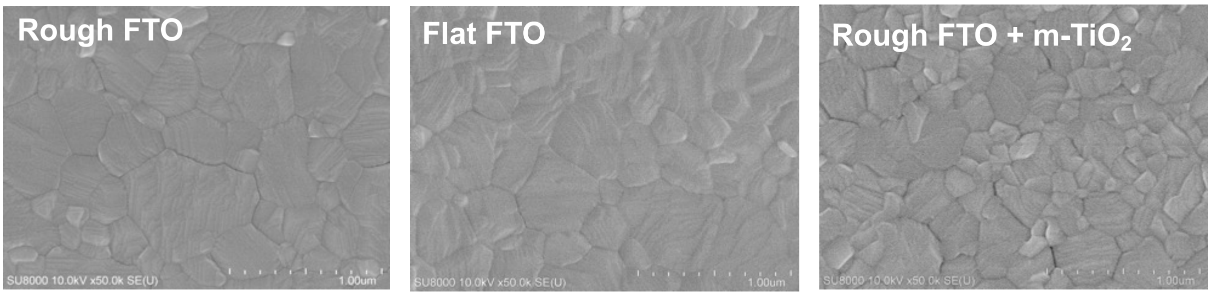

Fig. S3 Top view SEM images of perovskite films fabricated on each substrate.

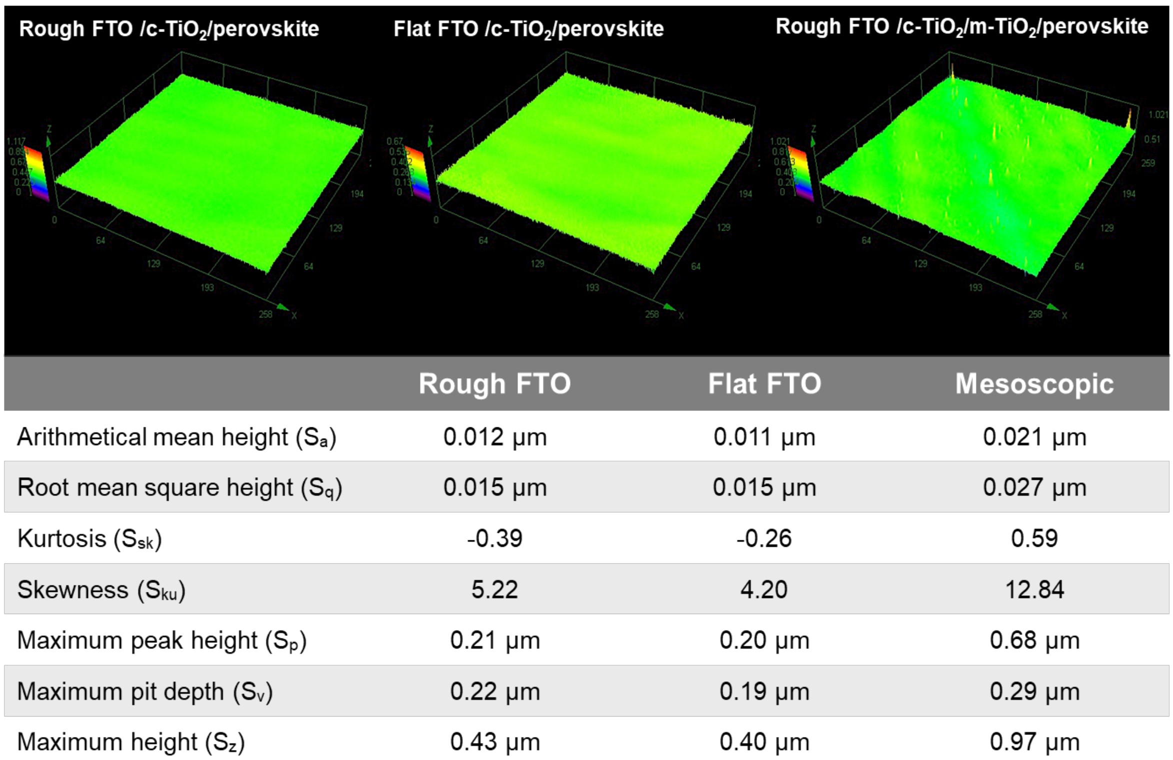

	Rough FTO	Flat FTO	Mesoscopic
Arithmetical mean height (S_a)	0.012 μm	0.011 μm	0.021 μm
Root mean square height (S_q)	0.015 μm	0.015 μm	0.027 μm
Kurtosis (S_{sk})	-0.39	-0.26	0.59
Skewness (S_{ku})	5.22	4.20	12.84
Maximum peak height (S_p)	0.21 μm	0.20 μm	0.68 μm
Maximum pit depth (S_v)	0.22 μm	0.19 μm	0.29 μm
Maximum height (S_z)	0.43 μm	0.40 μm	0.97 μm

Fig. S4 Surface roughness parameters of perovskite films fabricated on each underlayers. Perovskite film fabricated on compact TiO_2 deposited on Rough and Flat FTO is denoted as "Rough FTO" and "Flat FTO", respectively. Perovskite film fabricated on mesoporous TiO_2 and compact TiO_2 layer with Rough FTO is denoted as "Mesoscopic". Each parameter was measured with an area of 258 μm × 258 μm.

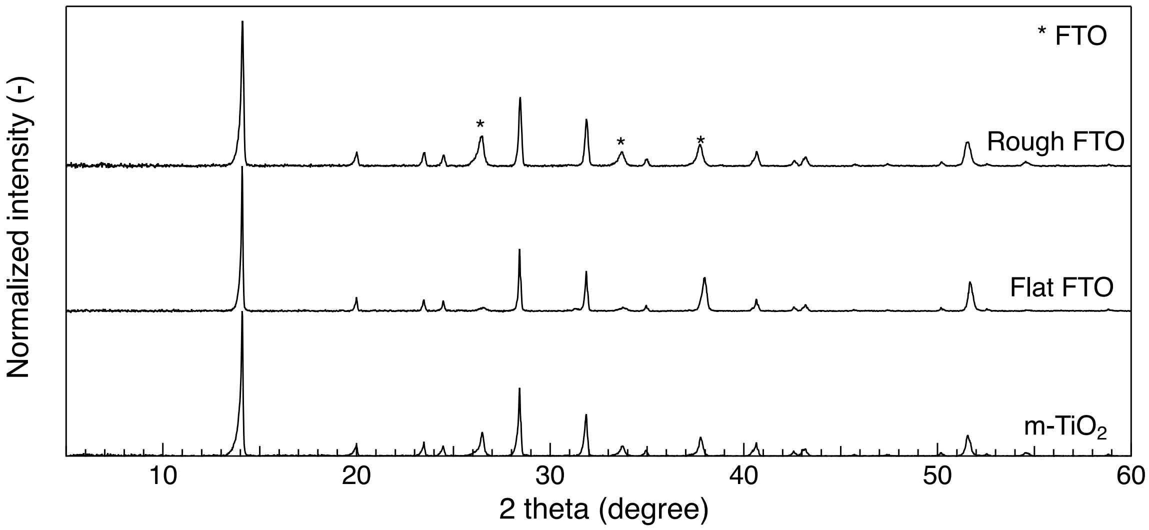

Fig. S5 X-ray diffraction patterns of perovskite films fabricated on each substrate.

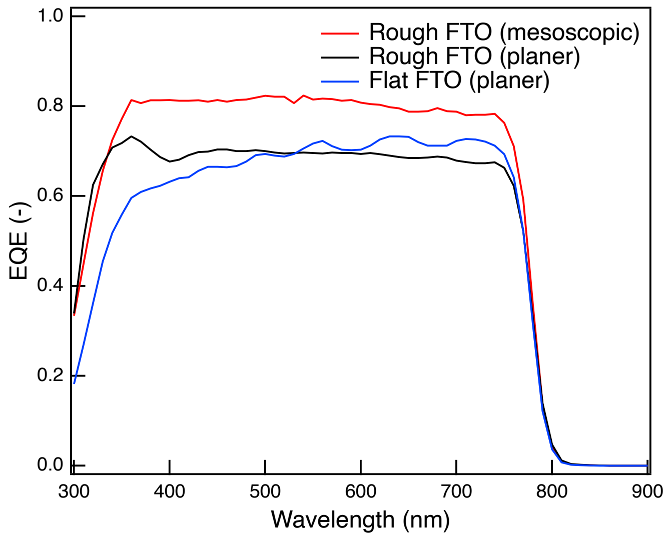

Fig. S6 EQE spectra of solar cell devices fabricated with a different surface morphology.

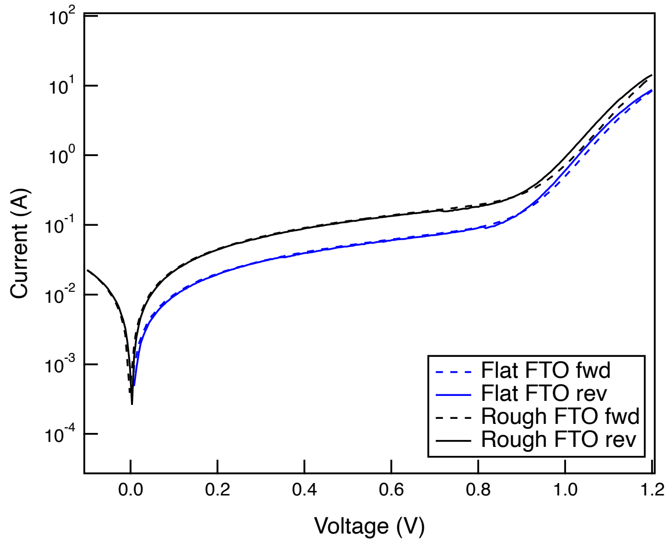

Fig. S7 Dark *J-V* curves of PSC devices fabricated with Rough and Flat FTO.

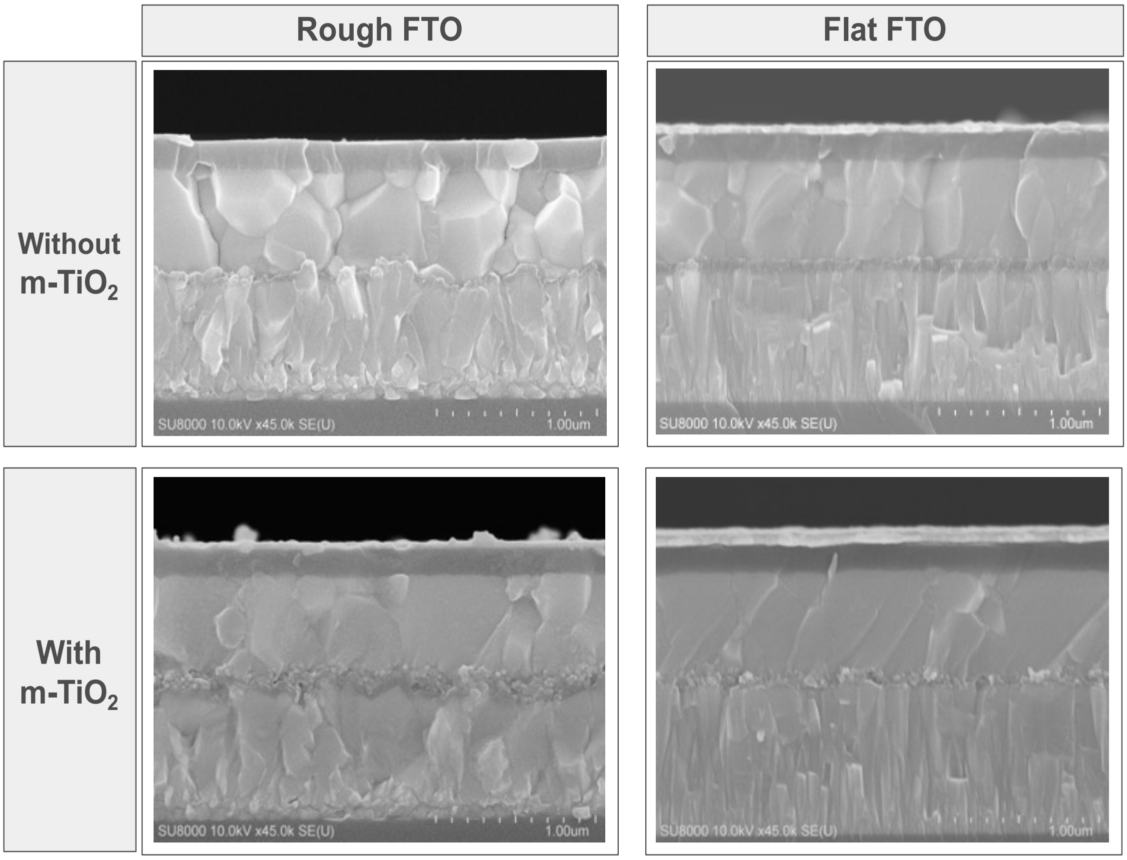

Fig. S8 Cross section SEM images of PSC devices fabricated on each substrate with planer hetero junction and mesoscopic structure.

Table S2 Photovoltaic parameters of the mesoscopic structured device fabricated with Rough and Flat FTO.

		J_{sc} ($mAcm^{-2}$)	V_{oc} (V)	FF (-)	η (%)	HI (-)
**Rough FTO**	Fwd	23.9	1.04	0.59	14.6	0.19
	Rev	23.9	1.08	0.70	18.0	
**Flat FTO**	Fwd	22.6	1.00	0.57	11.9	0.22
	Rev	22.6	1.05	0.69	16.3	

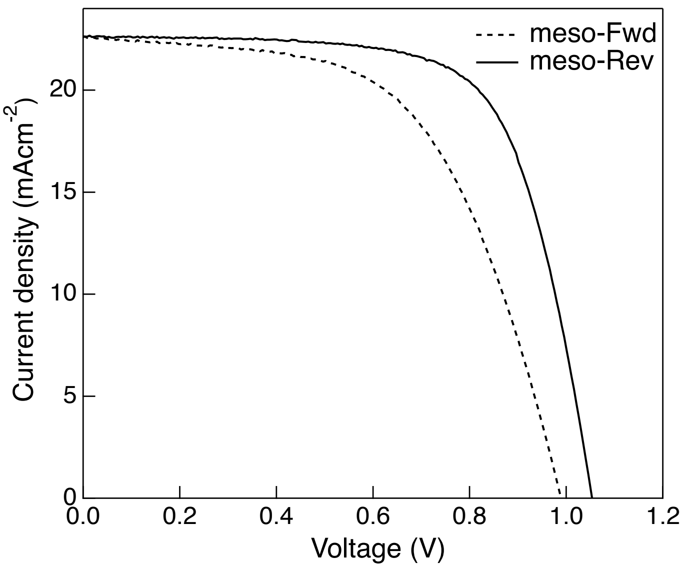

Fig. S9 *I-V* curves of the mesoscopic structured device fabricated with Flat FTO.
